# The relationship between psychological resilience and quality of life among the Chinese diabetes patients: the mediating role of stigma and the moderating role of empowerment

**DOI:** 10.1186/s12889-023-16927-7

**Published:** 2023-10-19

**Authors:** Yujin Mei, Xue Yang, Jiaofeng Gui, Yuqing Li, Xiaoyun Zhang, Ying Wang, Wenyue Chen, Mingjia Chen, Changjun Liu, Lin Zhang

**Affiliations:** 1 School of Nursing, Anhui Province, Wannan Medical College, 22 Wenchang West Road, Higher Education Park, Wuhu City, People’s Republic of China; 2 School of Marxism, Liaoning Province, Jinzhou Medical University, No.40, Section 3, Songpo Road, Linghe District, Jinzhou City, People’s Republic of China; 3 Department of Internal Medicine Nursing, School of Nursing, Wannan Medical College, 22 Wenchang West Road, Higher Education Park, Wuhu City, Anhui Province People’s Republic of China

**Keywords:** Psychological resilience, Stigma, Empowerment, Quality of life, Diabetes patients

## Abstract

**Background:**

Although some factors, such as stigma and empowerment, influence the complex relationship between psychological resilience and quality of life, few studies have explored similar psychological mechanisms among patients with diabetes. Therefore, this study explored the mediating role of stigma and the moderating role of empowerment in the psychological mechanisms by which psychological resilience affects quality of life.

**Methods:**

From June to September 2022, data were collected by multi-stage stratified sampling and random number table method. Firstly, six tertiary hospitals in Wuhu were numbered and then selected using the random number table method, resulting in the First Affiliated Hospital of Wannan Medical College being selected. Secondly, two departments were randomly selected from this hospital: endocrinology and geriatrics. Thirdly, survey points were set up in each department, and T2DM patients were randomly selected for questionnaire surveys. In addition, we used the Connor-Davidson Elasticity Scale (CD-RISC) to measure the psychological resilience of patients, and used the Stigma Scale for Chronic Illness (SSCI) to measure stigma. Empowerment was measured by the Diabetes Empowerment Scale (DES). Quality of Life was assessed by the Diabetes Quality of Life Scale (DQoL). We used SPSS (version 21) and PROCESS (version 4.1) for data analysis.

**Results:**

(1) Psychological resilience was negatively correlated with stigma and quality of life, and positively correlated with empowerment. Stigma was positively associated with empowerment and quality of life. Empowerment was negatively correlated with quality of life. (2) The mediation analysis showed that psychological resilience had a direct predictive effect on the quality of life, and stigma partially mediated the relationship; Empowerment moderates the first half of "PR → stigma → quality of life"; Empowerment moderates the latter part of "PR → stigma → quality of life."

**Conclusions:**

Under the mediating effect of stigma, psychological resilience can improve quality of life. Empowerment has a moderating effect on the relationship between psychological resilience and stigma, and it also has a moderating effect on the relationship between stigma and quality of life. These results facilitate the understanding of the relationship mechanisms between psychological resilience and quality of life.

## Introduction

Diabetes mellitus (DM) is a chronic noncommunicable disease with widespread prevalence [1]. In recent years, the prevalence of diabetes has increased dramatically with the change in lifestyle [2]. It has become a public health problem of wide concern and poses a threat to global health problems [3]. People with diabetes are prone to complications such as neurological and cardiovascular diseases and diabetic foot. In addition, psychological complications, such as anxiety and depression, are common, affecting psychosocial life and daily functioning and leading to poor quality of life(QoL) [4]. Relevant research results show that their QoL of diabetes patients is generally low, and the prolongation of diabetes is associated with a decline in QoL [5]. Impaired QoL can affect individual’s motivation to continue with the treatment, such as reluctance to be hospitalised or refusal to control blood glucose. Therefore, the study on the QoL of diabetes patients is particularly important.

QoL is a concept that comprehensively evaluates the merits of life and represents an individual's view of how well functioning is physically, psychologically, and socially [6]. QoL is a significant health outcome in its own right, representing the ultimate goal of all health interventions [7]. QoL is measured by physical and social functioning and perceived physical and mental health [8]. Studies have shown that the QoL of people with diabetes is reduced compared to those without diabetes [9]. Maintaining the QoL of people with diabetes is a decisive outcome variable for diabetes treatment [10]. It should be used as an essential quality indicator to evaluate the efficacy and effectiveness of therapeutic measures.

Up to now, QoL's relevant factors and influencing mechanisms have yet to be made clear. However, previous studies on the internal mechanism of quality of life have shown that one of the influencing factors is psychological resilience(PR) [11]. As an individual's ability to actively cope with difficult situations, PR can help individuals maintain a relatively stable physiological and psychological level in an unfavorable environment [12]. Individuals with higher levels of PR have been reported to have stronger positive social orientation abilities, can positively participate in social activities, and have a higher QoL than patients with the same disease [13, 14].

The potential mechanism of PR on QoL needs to be further explored, and stigma may play a mediating role in it. Stigma refers to negative emotional experiences such as negative self-cognition, self-blame, and self-depreciation caused by patients being discriminated against, excluded, and alienated by the public due to a certain disease [15]. Diabetes is usually stereotyped and considered to be caused by poor eating habits and lifestyles, which can easily lead to negative psychology, such as stigma [16, 17]. Several studies have shown that people with type 2 diabetes often feel stigma, with 17.1%-52% of the population feeling stigma for having the condition [18, 19]. An international study of Mexican patients with diabetes showed that 13.9% of this group had a stigma associated with the disease [20]. At the same time, studies had shown that stigma affects the level of PR and QoL of individuals and has a negative impact on the construction of individual PR and QoL [21, 22]. Stigma itself has a negative connotation and is closely related to the patient's negative emotions [23]. In the face of adversity or stressful events, diabetes with high levels of PR can accept the disease with a good attitude, actively face negative emotions, and have a lighter degree of stigma, which is conducive to the improvement of QoL [24]. Conversely, patients with low levels of PR tend to respond to difficulties in an avoidant manner, which increases the burden of disease and stigma and leads to a decline in QoL. Therefore, hypothesis 1 was proposed that PR had a direct predictive effect on the quality of life, and stigma partially mediated the relationship.

PR's direct and indirect effects on QoL may also be moderated by other variables, such as empowerment [25]. Empowerment refers to the process by which patients gain the necessary knowledge and self-awareness to influence their and others' behavior to improve their QoL [26]. Patient empowerment interventions can improve the capabilities of patients, giving them greater control over their disease-related parameters and lifestyle [27, 28]. Empowerment boosts confidence, awareness, and decision-making skills for physical and mental health and healthcare [29]. However, most diabetes patients have a lower level of PR due to a lack of disease knowledge, poor glycemic control, and increased disease burden, which further contributes to higher levels of stigma than the normal population [30]. Studies had shown that empowerment education interventions could effectively improve glycemic control, blood glucose levels, and QoL in people with diabetes [31]. Diabetes has many complications and poor recovery, and long-term drug treatment causes irreversible functional damage to the kidneys and other organs, which would aggravate the patient's experience of stigma and lead to a decline in QoL [32]. However, empowerment of diabetic patients can enable patients to correctly understand their disease, understand other harmful factors such as complications, help patients to build self-confidence, improve their motivation for treatment, alleviate the occurrence of disease stigma, and thus improve their QoL [33]. Therefore, hypothesis 2 proposed that empowerment moderates the relationship between PR, stigma and QoL.

**Fig. 1:**
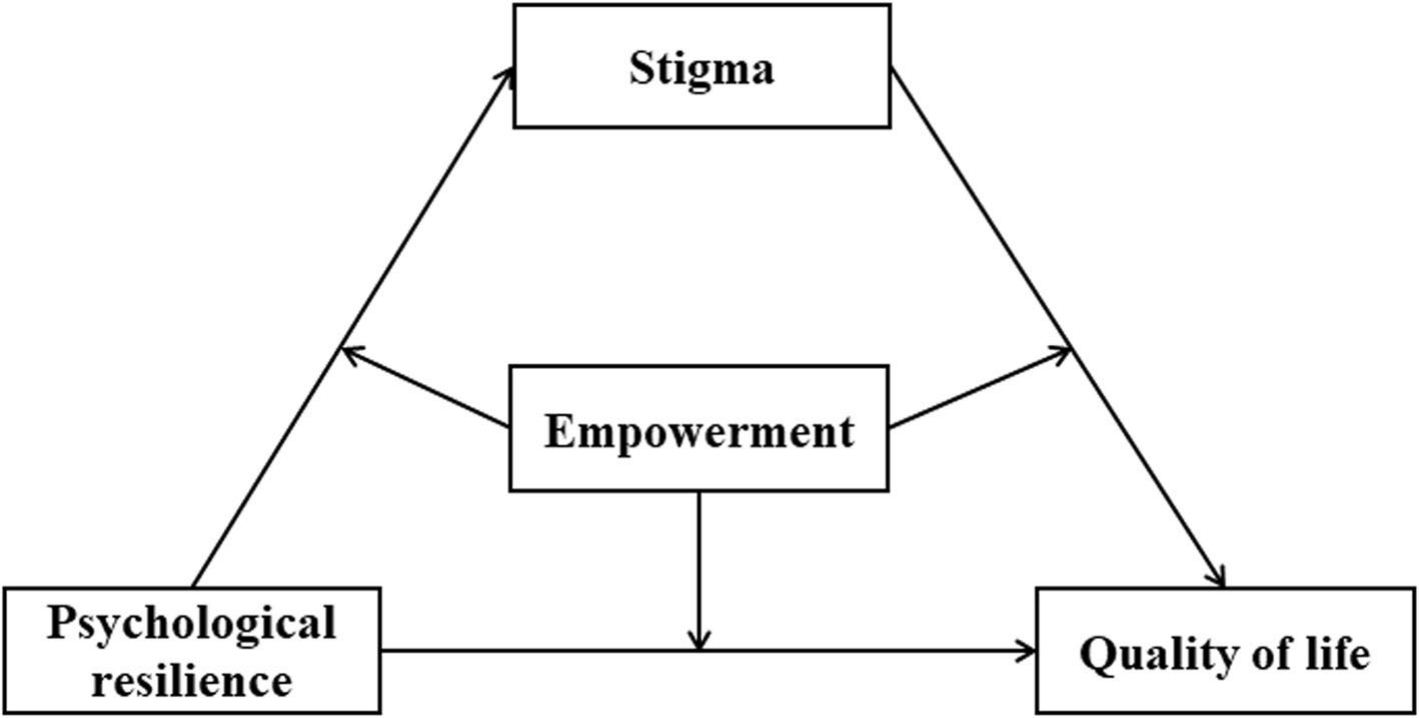
Hypothetical model of the relationships between PR, stigma, empowerment and QoL. PR, psychological resilience; QoL, quality of life

To further explore the relationship between PR, stigma, empowerment, and QoL, this study proposed a moderate mediation model to study the relationship between PR and QoL in patients with diabetes. This study proposed the following hypotheses:(H1) the mediating role of stigma between PR and QoL; (H2) Empowerment moderates the relationship between PR, stigma and QoL (Fig. 1).

## Materials and methods

### Study design and participants

From June to September 2022, this study used a multi-stage sampling method and random number table method to collect data in the Wuhu City, Anhui Province. Firstly, six tertiary hospitals in Wuhu were numbered and then selected using the random number table method, resulting in the First Affiliated Hospital of Wannan Medical College being selected. Secondly, two departments were strategically selected from this hospital: endocrinology and geriatrics. Thirdly, survey points were set up in each department, and diabetes patients were strategically selected for questionnaire surveys. The inclusion criteria were as follows: (i) All patients should meet the diagnostic criteria for DM established by the American Diabetes Association: 2-h PG ≥ 200 mg/dL (11.1 mmol/L) during OGTT [34]. (ii) Patients are conscious and have full mobility and cognitive ability. (iii) Patients are willing to cooperate and complete the questionnaire. Exclusion criteria are as follows: (i) the presence of severe mental disorders or intellectual problems, meaning patients who are completely unable to communicate or understand and think; (ii) severe diabetic complications or inability to take care of themselves, meaning that the patient is affected by the complications, which prevent him/her from communicating properly or that the patient is in a comatose state; and (iii) the existence of other serious illnesses, meaning that the patient has suffered from a serious cardiovascular disease, Serious infectious diseases, cancer, visual and hearing impairments due to diabetes complications, etc., which make the patient so weak that he/she is unable to take the questionnaire"; (iv) Pregnancy or other specific diabetes.

This study used structural equation modelling to test for moderated mediation effects, which, according to previous research, requires a sample size 10–15 times the number of variables [35]. A total of 21 variables (7 demographic items, 5 PR dimensions, 2 stigma dimensions, 3 empowerment dimensions, and 4 QOL dimensions) were included in this study. The final sample size was set at 231 ~ 347 to account for 10% non-responders. A total of 334 questionnaires were distributed and 329 valid questionnaires were returned, with a valid return rate of 95.85%. Finally, the actual sample size was 329, which met the requirements for analysis.

To reduce errors, relevant personnel was trained before the investigation to clarify communication skills and scoring standards. After obtaining the informed consent of the diabetes, the questionnaire was issued, and the patients answered by themselves. For illiterate patients, the investigators asked face-to-face and then filled out the questionnaire. All methods are implemented following the declaration of Helsinki.

### Measurements

#### Psychological resilience

The Conner-Davidson Resilience Questionnaire (CD-RISC) was developed by psychologists Professors Conner and Davidson in 2003 [36]. The CD-RISC contains 25 items on a five-point Likert scale ranging from 0 ("not at all true") to 4 ("almost always true"). The scale consists of 5 dimensions. The first dimension reflects high standards, resilience, and ability. The second dimension reflects dealing with emotions and believing in one's intuition. The third dimension reflects having a constructive attitude towards change and safe relationships. The fourth dimension is perceived control and the fifth dimension is mental strength. The Cronbach 's alpha value for this study scale was 0.861 [37].

#### Stigma

Rao developed the Stigma Scale for Chronic Illness (SSCI) to measure stigma in people with chronic illnesses [38]. It consists of 24 items and contains two dimensions: intrinsic stigma and extrinsic stigma. The first 13 items refer to internal stigma and ask about the respondent's own feelings of stigma. The next 11 items ask about the stigma the respondent feels due to external actions. Each item is rated from 0 (never) to 4 (always). The higher the score, the higher level of stigma. The Cronbach 's alpha value for this study scale was 0.829 [39].

#### Empowerment

Diabetes Empowerment Scale (DES) was compiled by Anderson R and Funnell MM in 2000 [40]. The scale includes three dimensions of psychosocial management of diabetes, assessment of dissatisfaction and readiness for change, and setting and achieving diabetes goals, with a total of 28 items. The Likert 5-level score was adopted. The scale ranges from 0 (strongly disagree) to 4 (strongly agree). The higher the score, the higher empowerment ability. The Cronbach's alpha value for this study scale was 0.960 [41].

#### Quality of life

Diabetes Quality of Life Scale (DQoL) was developed by the UK Diabetes Control and Complications Trial Research Group in 1988 [42], the scale includes four dimensions of satisfaction, impact, diabetes-related worry, and social/occupational worry, with a total of 15 items, using a Likert 5 scale, from 0 (never) to 4 (always) and 0 (very satisfied) to 4 (very dissatisfied) respectively. A lower score indicates a better QoL. The Cronbach's alpha value for this study scale was 0.920 [43].

### Statistical analyses

Harman single factor test was used for exploratory factor analysis of all the questionnaire items. The results showed that there were 25 factors with eigenvalues greater than 1. The first factor explained only 18.913% of the variance, which was less than 40% critical standard, suggesting that there was no common methodological bias.

We used SPSS 23.0 to accomplish all the statistical analyses. Firstly, we calculated general and controlled variables for descriptive statistics and bivariate correlations. Secondly, we used Hayes' (2013) PROCESS macro (Model 4) to evaluate the mediating effect of stigma. Finally, we analyzed the moderator–mediator model with Hayes's PROCESS macro (Model 8) (2013). All the continuous variables were standardized, and the interaction terms were calculated from these standardized scores. The bootstrap method produces 95% bias-corrected CIs for these effects from 5000 re-sample of the data. CIs that do not contain zero indicate a significant effect.

## Results

### Descriptive statistics

Table 1 shows the demographic characteristics of the subjects and a univariate analysis of the QoL scores with different characteristics, relatively high, which represents the low quality of life of diabetic patients; PR score of 33.73 ± 13.71, which is low compared to the score of normal population, indicating that diabetic patients have a low level of PR; stigma score of 36.22 ± 16.39, which shows that diabetic patients have a high level of stigma; and empowerment score of 37.18 ± 13.16, which is a relatively low score, indicating that diabetic patients need to receive some empowerment education. Among 329 diabetes patients, 198 (60.2%) were males, and 131 (39.8%) were females. Patients with diabetes range in age from 45 to 95 years. The difference of monthly income and SMBG in diabetes patients QoL scores were statistically significant (*P* < 0.05). Most diabetes patients (72.9%) had a secondary school education or below. Only 8.5 percent of diabetes were able to perform SMBG regularly, and more than a third (37.4 percent) of diabetes had a monthly income of less than 1,000 yuan.

**Table 1:** Univariate analysis of quality of life of diabetic patients with different characteristics (*n* = 329)

Variables	Group	N (%)	Mean ± SD	*F/t*	*P*
Gender	Male	198(60.2)	33.08 ± 7.19	0.099	0.753
	Female	131(39.8)	34.31 ± 7.45		
Education level	Middle school or less	240(72.9)	34.16 ± 6.93	2.937	0.054
	High or technical secondary school	49(14.9)	31.96 ± 7.65		
	Junior college or university	40(12.2)	32.00 ± 8.64		
Monthly income	Less than 1000 CNY	123(37.4)	35.14 ± 7.24	3.942	0.009
	1000–3000 CNY	55(16.7)	32.73 ± 7.18		
	3000–5000 CNY	77(23.4)	33.52 ± 6.57		
	Above 5000 CNY	74(22.5)	31.65 ± 7.80		
Course of the disease	< 5 years	101(30.7)	34.02 ± 6.98	1.115	0.343
	5–10 years	86(26.1)	32.57 ± 7.25		
	11–20 years	93(28.3)	33.39 ± 7.83		
	> 20 years	49(14.9)	34.76 ± 6.99		
Treatment	Take the medicine orally only	150(45.6)	33.90 ± 7.67	0.401	0.670
	With insulin alone	89(27.1)	33.02 ± 6.76		
	Medication combined with insulin	90(27.4)	33.57 ± 7.25		
SMBG	Never monitoring	106(32.2)	36.29 ± 6.30	18.137	< 0.001
	No law	195(59.3)	32.90 ± 7.14		
	Regular monitoring	28(8.5)	27.96 ± 7.97		
Severe hypoglycemia	Yes	78(23.7)	33.18 ± 7.18	0.009	0.923
	NO	251(76.3)	33.69 ± 7.36		

For dichotomous variables, independent samples t-tests were used, and one-way ANOVA was used for tertiary or multicategory variables

### Bivariate correlation analysis

The mean value, standard deviation, and correlation among variables are shown in Table 2. QoL scores were 33.57 ± 7.31 points. The results showed that PR was negatively correlated with stigma (*r* =  − 0.325, *P* < 0.01) and QoL (*r* =  − 0.503, *P* < 0.01), and positively correlated with empowerment (*r* = 0.434, *P* < 0.01). Stigma was positively correlated with QoL (*r* = 0.726, *P* < 0.01)and empowerment(*r* = 0.045, *P* < 0.01). Empowerment was negatively correlated with QoL (*r* =  − 0.199, *P* < 0.01).

**Table 2:** Descriptive statistics and correlations among variables (*n* = 329)

Variables	Mean	SD	1	2	3	4
1 PR	33.73	13.71	1			
2 Stigma	36.22	16.39	− 0.325^**^	1		
3 Empowerment	37.18	13.16	0.434^**^	0.045^**^	1	
4 QoL	33.57	7.31	− 0.503^**^	0.726^**^	− 0.199^**^	1

Using bivariate correlation analysis

^**^: *P* < 0.01. *PR* Psychological resilience, *QoL* Quality of Life

### Mediation analysis

To investigate hypothesis 1, after controlling the demographic variables of personal monthly income and SMBG, we used the PROCESS 4.1 macro proposed by Hayes (Model 4) to test the mediating effect of stigma on the relationship between PR and QoL (Table 3). The results showed that PR was negatively correlated with QoL (*β* =  − 0.151, *P* < 0.001). PR was negatively correlated with stigma *(β* =  − 0.378, *P* < 0.001). Stigma was positively correlated with QoL (*β* = 0.270, *P* < 0.001). We tested the PR indirect effect on the QoL (*β* =  − 0.102, *SE* = 0.022, 95% CI = [− 0.145, − 0.056]) and the direct effect (*β* =  − 0.151, SE = 0.021, 95%CI = [− 0.305, − 0.202]). The results showed that stigma partially mediated the relationship between PR and QoL (Table 4). Indirect and direct effect accounted for 40.32% and 59.68% of the total effect, respectively.

**Table 3:** Testing the mediation effect of PR on QoL

Variables	Stigma				QoL			
	*β*	*SE*	*t*	95%CI	*β*	*SE*	*t*	95%CI
Monthly income	1.092	0.748	1.460	− 0.380, 2.565	− 0.1119	0.227	− 0.527	− 0.565, 0.326
SMBG	− 6.421	1.426	− 4.502^***^	− 9.227, − 3.616	− 1.269	0.443	− 2.861^**^	− 2.141, − 0.396
PR	− 0.378	0.065	− 5.813^***^	− 0.507, − 0.250	− 0.151	0.021	− 7.333^***^	− 0.192, − 0.111
Stigma					0.270	0.017	16.112^***^	0.237, 0.303
*R*^*2*^			0.161				0.617	
*F*			20.789				130.649	

Using Hayes’ (2013) PROCESS macro (Model 4) in the SPSS

^*^: *P* < 0.05, **: *P* < 0.01, ***: *P* < 0.001. *SMBG* Self Monitor Blood Glucose, *PR* Psychological resilience, *QoL* Quality of Life

**Table 4:** Results for effects of PR on QoL with stigma as a mediator

	Effect	BootSE	BootLLCI	BootULCI	Relative effect size
Indirect effect	− 0.102	0.022	− 0.145	− 0.056	40.32%
Direct effect	− 0.151	0.021	− 0.192	− 0.111	59.68%
Total effect	− 0.253	0.026	− 0.305	− 0.202	100.00%

Using Hayes' (2013) PROCESS macro (Model 4) in the SPSS

### The moderation analyses

To test hypotheses 2 and 3, we use the PROCESS macro proposed by Hayes (Model 8) to test the moderated mediation. In particular, the parameters of the two models are estimated. In Model 1, we estimated the moderating effect of empowerment on the relationship between PR and stigma. In Model 2, we estimate the moderating effect of empowerment on the relationship between stigma and QoL.

**Fig. 2:**
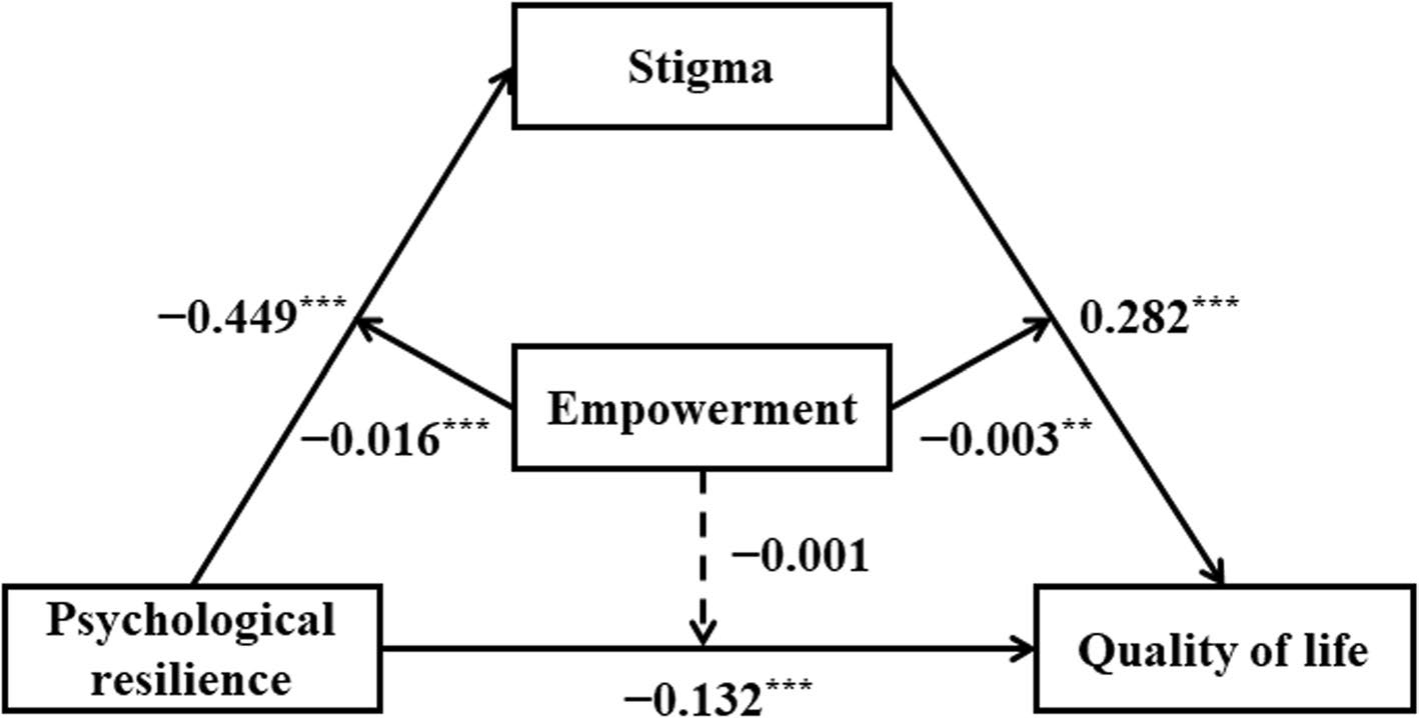
The moderated mediation model. *p < 0.05, **p < 0.01, ***p < 0.001

As shown in Table 5, Model 1 reveals the main effect of PR on stigma (*β* =  − 0.449, *SE* = 0.067, 95%CI = [− 0.580, − 0.318]), while empowerment plays a moderating role (*β* =  − 0.016, *SE* = 0.003, 95%CI = [− 0.022, − 0.010]). Model 2 showed that stigma had a significant effect on quality of life (*β* = 282, *SE* = 0.018, 95%CI = [0.247, 0.317]), and empowerment had a moderating effect (*β* =  − 0.003, *SE* = 0.001, 95%CI = [− 0.006, − 0.001]).The results showed that PR had a significant effect on the QoL (*β* =  − 0.132, *SE* = 0.023, 95%CI = [− 0.177, − 0.088]), and empowerment had no moderating effect (*β* =  − 0.001, *SE* = 0.001, 95% CI = [− 0.003, 0.001]). Therefore, hypotheses 2 and 3 were partially supported. The final mediation model is shown in Fig. 2.

**Table 5:** Results of the moderated mediation model analysis

Variables	Model 1(Stigma)				Model 2(QoL)			
	*β*	*SE*	*t*	95%*CI*	*β*	*SE*	*t*	95%*CI*
Monthly income	0.718	0.702	1.023	− 0.663, 2.099	− 0.004	0.223	− 0.019	− 0.444, 0.435
SMBG	− 6.559	1.334	− 4.915^***^	− 9.184, − 3.933	− 0.902	0.443	− 2.035^*^	− 1.773, − 0.030
PR	− 0.449	0.067	− 6.732^***^	− 0.580, − 0.318	− 0.132	0.023	− 5.811^***^	− 0.177, − 0.088
Stigma					0.282	0.018	16.034^***^	0.247, 0.317
Empowerment	0.449	0.072	6.234^***^	0.307, 0.591	− 0.085	0.024	− 3.499^***^	− 0.133, − 0.037
PR × Empowerment	− 0.016	0.003	− 5.364^***^	− 0.022, − 0.010	− 0.001	0.001	− 0.816	− 0.003, 0.001
Stigma × Empowerment					− 0.003	0.001	− 2.870^**^	− 0.006, − 0.001
*R*^*2*^			0.271				0.638	
*F*			23.955				80.788	

Using Hayes’ (2013) PROCESS macro (Model 8) in the SPSS

^*^: *P* < 0.05, **: *P* < 0.01, ***: *P* < 0.001. *SMBG* Self Monitor Blood Glucose, *PR* Psychological resilience, *QoL* Quality of Life

**Fig. 3:**
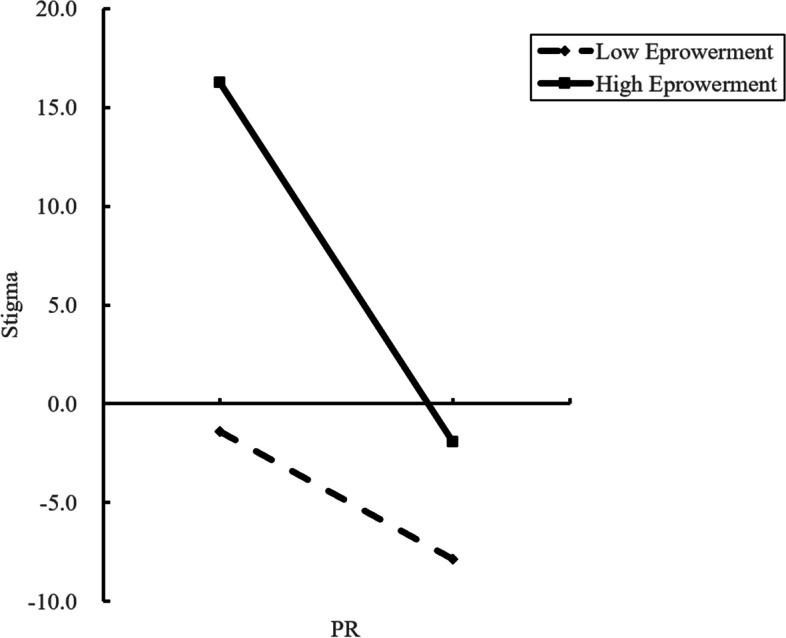
The moderating role of empowerment between psychological resilience and stigma

Figure 3 visually shows how the impact of PR on stigma is moderated by empowerment. A simple slope test showed that for high-empowered diabetes patients (*Z* = 1), there was a significant downward trend in stigma as the level of PR increased (*β* =  − 0.157,* P* < 0.001). One standard deviation increase in PR was associated with a 0.157 standard deviation decrease in total stigma. The higher the level of PR, the lower the level of stigma. However, PR did not predict stigma in low-empowerment diabetes patients.

**Fig. 4:**
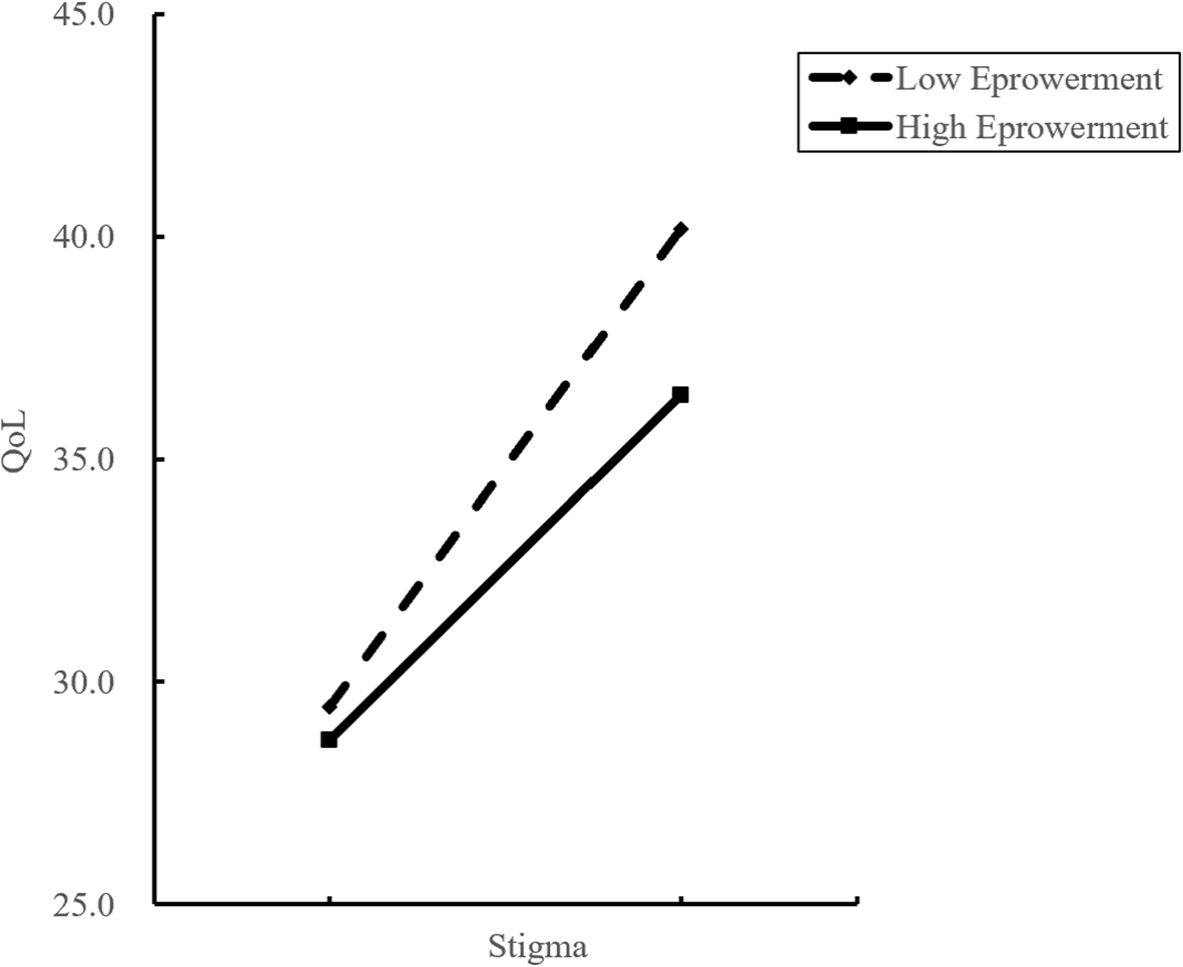
The moderating role of empowerment between stigma and quality of life scores

Figure 4 shows how empowerment moderates the relationship between stigma and QoL. The simple slope test showed that for high-empowered diabetes patients (*Z* = 1), there was a significant upward trend in the QoL scores (*β* = 0.237, *P* < 0.001) as the level of stigma increased, and one standard deviation increase in stigma was associated with a 0.237 standard deviation increase in QoL scores, the higher QoL score, the worse quality of life. For low-empowerment diabetes (*Z* =  − 1), QoL scores increased significantly with the increase in stigma (*β* = 0.327,* P* < 0.001), and an increase of one standard deviation in stigma was associated with a 0.327 standard deviation increase in QoL scores, larger than the increase in high-empowerment diabetes.

## Discussion

This study constructed a moderated mediation model to certify that PR affects QoL through stigma, and empowerment moderates the first half of the mediated pathway by which PR affects QoL through stigma; Empowerment moderates the second half of the mediated pathway by which PR affects QoL through stigma. Moderating analysis showed that PR significantly impacted stigma at high-level empowerment of diabetes patients, and stigma significantly impacted QoL at high or low empowerment of diabetes patients.

This study found that after controlling variables, PR still had a significant negative predictive effect on the QoL scores of diabetes patients. Higher PR levels are associated with lower QoL scores, better quality of life in diabetic patients. Meanwhile, in previous studies [44], the PR score of normal population was 68.82 ± 12.97, however, in our study the PR score of diabetes patients was only 33.73 ± 13.71, which was much lower than the normal population, and this phenomenon suggests that the PR level of diabetes patients is low compared to the normal population, which is consistent with the findings of previous studies have shown that PR is related to individual social adaptability and the QoL, has many positive effects on maintaining the function, subjective well-being, and improving QoL, and plays an important role in defense against negative events [45, 46]. Diabetes is a chronic disease that requires ongoing medical management to reduce the risk of acute and chronic complications and improve QoL [47, 48]. However, patients with diabetes may be deeply affected by poor blood sugar control, long-term diet and drug control symptoms, and a variety of complications of physical damage, so that patients feel physically and mentally exhausted, QoL seriously decreased, easy to cause negative emotions [49–51]. A study has shown that PR is an important predictor of QoL, reducing anxiety and depression and enabling individuals to achieve a higher QoL [52].

This study found that stigma mediated the relationship between PR and QoL in patients with diabetes. The impact of PR on the QoL of patients with diabetes is realized through a direct path on the one hand and an indirect path through influencing stigma on the other hand. Studies had shown that PR was an essential predictor of post-stress growth [53]. If people can maintain good PR after the illness. They are more inclined to face the disease positively and optimistically, thus showing less stigma experience, weakening the negative impact of the disease, and improving the QoL [54]. At the same time, the stigma of patients with diabetes also affects blood sugar control. When blood sugar is not controlled, patients will feel anxious, the stigma will be increased, and the QoL will be decreased [55, 56]. In addition, such diseases are often considered to be the result of unhealthy lifestyles, such as poor diet and lack of exercise, and are susceptible to social prejudice and discrimination, leading to reduced contact with the outside world and severely reducing the QoL of patients [57, 58]. Therefore, to improve the QoL of patients with diabetes, attention should be paid to the direct impact of PR on QoL and the indirect impact of PR on QoL through stigma.

This study found that empowerment significantly moderates the first half of the path by which PR affects the QoL through stigma. The impact of PR on stigma was significant for high-empowered diabetes patients. For low-level empowered diabetes, the impact of PR on stigma was not significant. High empowerment of diabetes patients, better understanding of their own disease and control effect [59]. Furthermore a better empowerment can reduce the disease burden, and protect the physical and mental health of patients, which is conducive to the reduction of stigma level [60]. The results of the study suggest that there is a need to focus on the level of empowerment of people with diabetes along with measures to improve the level of PR of people with diabetes.

In addition, the present study found that empowerment significantly moderates the second half of the path through which PR affects the QoL through stigma. Specifically, as stigma increased in low-empowerment diabetes patients, QoL scores increased more than in high-empowerment diabetes patients. The higher the QoL score, the worse quality of life. It can be seen that the quality of life of people with low levels of empowerment diabetes is more affected by stigma and more prone to a reduced quality of life due to increased stigma [61, 62]. The study suggests that when improving the quality of life of people with diabetes, attention should also be paid to the level of stigma and empowerment of patients.

There are several limitations to this study. Firstly, we could not make any causal inferences about the observed associations due to the study's cross-sectional design. Future research should use longitudinal studies to better define the pathways in our theoretical model. Secondly, although self-reporting has been widely used in the literature, this data collection method has inherent disadvantages, such as being highly subjective, inevitably leading to some bias in the data. Future research should include multiple data collection methods to cross-check and obtain more objective and accurate data.

## Conclusion

This study examined the relationship between PR and QOL in Chinese diabetic patients using a moderated mediator model. There was a significant negative correlation between PR and QOL scores, with stigma partially mediating the relationship between PR and QOL. The model was moderated by empowerment, and PR had a much greater effect on stigma in patients with higher levels of empowerment. At the same time, the effect of stigma on QOL was also much greater in patients with lower levels of empowerment.

## Data Availability

The datasets used and analyzed during the current study are available from the corresponding author on reasonable request.

## Abbreviations

CD-RISC: Conner-Davidson Resilience Scale
SSCI: Stigma Scale for Chronic Illness
DQoL: Diabetes Quality of Life Scale
DES: Diabetes Empowerment Scale
CIs: Confidence intervals
DM: Diabetes Mellitus
SMBG: Self Monitor Blood Glucose
PR: Psychological Resilience
QoL: Quality of Life
ANOVA: One-way Analysis of Variance
OGTT: Oral Glucose Tolerance Test
2-h PG: 2-H Plasma Glucose
